# Knowledge and perceptions about Zika virus in a Middle East country

**DOI:** 10.1186/s12879-017-2603-6

**Published:** 2017-07-26

**Authors:** Sohaila Cheema, Patrick Maisonneuve, Ingmar Weber, Luis Fernandez-Luque, Amit Abraham, Hekmat Alrouh, Javaid Sheikh, Albert B. Lowenfels, Ravinder Mamtani

**Affiliations:** 1 Institute for Population Health, Weill Cornell Medicine-Qatar, Education City, Qatar Foundation, P.O. Box: 24144, Doha, Qatar; 2 Division of Epidemiology and Biostatistics, European Institute of Oncology, Milan, Italy; 3 Qatar Computing Research Institute, Doha, Qatar; 4 Office of the Dean, Weill Cornell Medicine-Qatar, Education City, Qatar Foundation, P.O. Box 24144, Doha, Qatar; 5 Department of Surgery and Department of Family Medicine, New York Medical College, Valhalla, NY USA

**Keywords:** Zika virus, Infection, Flavivirus, Education, Survey, Epidemiology, Qatar, Middle East

## Abstract

**Background:**

Zika virus, an emerging serious infectious disease, is a threat to persons living or travelling to regions where it is currently endemic, and also to contacts of infected individuals. The aim of this study was to assess knowledge about this new public health threat to persons residing in a Middle Eastern country.

**Methods:**

We conducted a survey at several international universities in Qatar to assess knowledge and awareness about this disease. An adapted version of the survey was also conducted using online channels from Qatar.

**Results:**

The median age of the 446 participants, was 25 years, 280 (63%) were females, and 32% were from Gulf Cooperation Council (GCC) or other Middle East countries. Based upon their knowledge about availability of a vaccine, role of mosquitoes and other modes of transmission, and disease complications, we classified respondent’s knowledge as “*poor*” (66%), “*basic*” (27%) or “*broad*” (7%). Forty-five (16%) persons with poor knowledge considered themselves to be well-informed.

**Conclusions:**

This report from a sample of persons associated with Middle East educational complex, reveals inadequate knowledge about Zika virus, a serious emerging infectious disease. Although few cases have been reported from the region, future cases are possible, since this area is a transit hub connecting currently infected regions to North America, Europe and Asia. As a preventive measure, an educational program about Zika virus would be valuable, especially for individuals or family members travelling to afflicted regions.

## Background

Transmission of Zika virus has been reported in multiple countries throughout the world [1]. Although the disease is usually mild, because of serious complications such as microcephaly and Guillain-Barre disease, Zika virus has been previously declared a Public Health Emergency of International Concern [2–4]. There is no prior knowledge of a mosquito borne illness, which has been associated with birth defects or capable of sexual transmission; Zika virus infection thus presents an unprecedented threat [5].

Due to the threat it presents, all nations around the world need to be vigilant about Zika virus’s potential to spread to remote regions including the Middle East [6], where potential primary vector species (*Aedes albopictus* and *Aedes aegypti* mosquitoes) are present [7, 8]. Qatar is a high-income Gulf Cooperation Council (GCC) country located on the northeastern coast of the Arabian Peninsula and harbors the primary vector species, *Aedes aegypti* for Zika virus [7]. Qatar Airways, the national carrier for the State of Qatar has daily flights to and from Sao Paulo in Brazil, Miami in Florida, and Argentina, some of the countries/territories where active transmission of Zika virus has been confirmed and documented. Additionally, Qatar Airways operates three flights a day to Singapore, another country which has been added to the watch list where currently active Zika virus transmission is ongoing. Emirates and Etihad Airlines, two other GCC nation carriers also have an extensive network of flight destinations to countries/territories where Zika virus cases have been reported. This also increases the likelihood of importation of Zika virus cases to the region. Additionally, most of the GCC countries have large expatriate workers originating in countries where other flavivirus infections have been reported; this could potentially lead to dissemination and spread of the Zika virus to the region.

Successful disease preventive programs depend upon public awareness of risk factors and disease characteristics. This study aims to assess the knowledge about the Zika virus and its risks among residents in Qatar.

## Methods

### Study participants

We conducted a survey in Education City, which houses a multinational population of approximately 5750 students, faculty, and staff who come from countries around the world to study and work in Qatar. The sample size was estimated by contacting Student Affairs and Human Resource Divisions of the institutions housed in Education City. The sample size of 396 was estimated using a margin of error of 5% and 95% confidence interval levels to appropriately represent the population in Education City. An additional 10% of the estimated sample size was recruited to account for attrition and incomplete responses. An English language survey adapted from existing literature was used to gather socio-demographic information, and questions evaluating the knowledge and perceptions towards Zika virus, such as communicability, availability of vaccine, symptoms and treatment. No personally identifiable information was collected. Trained personnel from the Division of Global and Public Health at Weill Cornell Medicine – Qatar approached potential subjects in person to conduct the self-administered survey. All recruited participants were 18 years or above. Data was collected from August – September 2016 and entered in an ad-hoc database.

### Survey questionnaires

An online version of the survey was posted on the internet along with an Arabic translation on the Qatar-based websites QatarLiving.com and iLoveQatar.net to reach a wider sample of the general public. Participants were also recruited via online advertising on Google search results and through the social media channel of Facebook. As part of the survey, all participants had to check off that they were above 18 years of age and resident in Qatar at the time of participation. The online recruitment was open for the same period as the self-administered survey version.

The self-administered survey questionnaires (supplementary material) included a series of questions about participant demographic characteristics (gender, age, nationality, marital status, education, role, institution), general information about Zika virus (source of information, existence of a vaccine to prevent Zika, mode of transmission of Zika, individuals at risk of Zika infection), personal knowledge about Zika and questions related to participants’ travel to endemic countries.

Based on the correctness of responses to questions related to facts about Zika virus, we classified participants’ general knowledge about Zika virus as: “*poor*” when responders didn’t know that there is currently no vaccine for Zika virus and/or that the disease is transmitted by infected mosquitoes and/or that anyone could get Zika virus; “*basic*” when responders knew that there is currently no vaccine for Zika virus, that the disease is transmitted by infected mosquitoes and that anyone could get Zika virus; and “*broad*” when in addition, responders knew that the disease could be transmitted through sexual intercourse, blood transfusion or during pregnancy, and that if a pregnant woman has Zika virus, there are risks for her baby/fetus.

### Statistical analysis

We used simple descriptive statistics (frequencies and percentage) to describe responses to the questionnaire. The Chi-square test was used to test for differences between groups. We used univariate and multivariable logistic regression to investigate participants’ characteristics that are associated with “basic” or “broad” knowledge about Zika virus, taking those with “poor” knowledge as reference group. Only factors associated with knowledge status at univariate analysis were included in the multivariable logistic regression model, to identify independent predictors. For nationality, we used participants from GCC and other middle east countries as reference group, we grouped participants from Asia and Africa, and those from Europe, North America, Australia and New Zealand. For participants’ role, we used students who represent the largest group as reference. For institution, a preliminary analysis (data not shown) revealed that knowledge was similar for all those non affiliated to a school of medicine or art, and this joint group was used a reference. Analyses were performed with SAS version 9.2 (Cary, NC). All *p*-values were two-sided. *P*-values <0.05 were considered statistically significant.

## Results

### Cross sectional survey conducted in Education City, Doha, Qatar and online survey

The response rate for the online survey was 94%. Demographic characteristics of the 446 participants, 161 (36.1%) males and 280 (62.8%) females, are presented in Table 1 **.** Fifty (11.2%) participants responded to the online survey and 396 (88.8%) to the survey administered in the education city, including 191 (42.8%) students, 162 (36.3%) staff and 42 (9.4%) faculty members. The median age of the participants was 25 years, ranging from 18 to 71 years; 256 (57.4%) were single. Sixty five (14.6%) participants are originating from a GCC country including Qatar, 76 (17.0%) from other Middle East countries; 30 (6.7%) from Africa, 131 (29.4%) from Asia, 31 (7.4%) from Europe and 81 (18.2%) from North America, Australia or New Zealand.

**Table 1:** Demographic characteristics of participants to the Zika virus survey

Variable	Category	All	Female	Male	Student	Staff	Faculty	Online Survey
		N	%	N	%	N	%	*N*
	All participants	446 (100)	280 (100)	161 (100)	191 (100)	162 (100)	42 (100)	50 (100)
Gender	Female	280 (62.8)	280 (100)	-	132 (69.1)	113 (69.8)	16 (38.1)	19 (38.0)
	Male	161 (36.1)	-	161 (100)	56 (29.3)	48 (29.6)	26 (61.9)	31 (62.0)
	Missing	5 (1.1)	0 (0.0)	0 (0.0)	3 (1.6)	1 (0.6)	0 (0.0)	0 (0.0)
Age	Median (range) years	25 (18–71)	23 (18–60)	30 (18–71)	20 (18–35)	34 (21–62)	37 (18–71)	33 (18–58)
	Less than 20	88 (19.7)	66 (23.6)	21 (13.0)	84 (44.0)	0 (0.0)	1 (2.4)	3 (6.0)
	20–29	163 (36.5)	106 (37.9)	56 (34.8)	102 (53.4)	43 (26.5)	2 (4.8)	16 (32.0)
	30–39	98 (22.0)	58 (20.7)	40 (24.8)	3 (1.6)	67 (41.4)	19 (45.2)	9 (18.0)
	40 or more	76 (17.0)	36 (12.9)	39 (24.2)	0 (0.0)	44 (27.2)	16 (38.1)	16 (32.0)
	Missing	21 (4.7)	14 (5.0)	5 (3.1)	2 (1.0)	8 (4.9)	4 (9.5)	6 (12.0)
Marital status	Single	256 (57.4)	169 (60.4)	84 (52.2)	181 (94.8)	41 (25.3)	7 (16.7)	27 (54.0)
	Married	171 (38.3)	96 (34.3)	74 (46.0)	8 (4.2)	114 (70.4)	30 (71.4)	19 (38.0)
	Divorced	9 (2.0)	7 (2.5)	2 (1.2)	1 (0.5)	3 (1.9)	3 (7.1)	2 (4.0)
	Widow/Widower	3 (0.7)	3 (1.1)	0 (0.0)	0 (0.0)	2 (1.2)	0 (0.0)	1 (2.0)
	Missing	7 (1.6)	5 (1.8)	1 (0.6)	1 (0.5)	2 (1.2)	2 (4.8)	1 (2.0)
Nationality	GCC countries	65 (14.6)	55 (19.6)	9 (5.6)	41 (21.5)	10 (6.2)	2 (4.8)	12 (24.0)
	Middle East	76 (17.0)	45 (16.1)	31 (19.3)	37 (19.4)	27 (16.7)	6 (14.3)	6 (12.0)
	Africa	30 (6.7)	20 (7.1)	10 (6.2)	19 (9.9)	8 (4.9)	2 (4.8)	1 (2.0)
	Asia	131 (29.4)	90 (32.1)	41 (25.5)	60 (31.4)	57 (35.2)	4 (9.5)	10 (20.0)
	Europe	31 (7.0)	14 (5.0)	19 (11.8)	4 (2.1)	13 (8.0)	9 (21.4)	7 (14.0)
	North America/Australia/New Zealand	81 (18.2)	41 (14.6)	38 (23.6)	22 (11.5)	38 (23.5)	15 (35.7)	6 (12.0)
	Missing	30 (6.7)	15 (5.4)	13 (8.1)	8 (4.2)	9 (5.6)	4 (9.5)	8 (16.0)
For students, staff or faculty members only: Which institution do you belong to?	All institutions	396 (100)	261 (100)	130 (100)	191 (100)	162 (100)	42 (100)	
	VCU-Q (Art, design)	48 (12.1)	40 (15.3)	7 (5.4)	44 (23.0)	3 (1.9)	1 (2.4)	-
	WCM-Q (Medicine)	74 (18.7)	54 (20.7)	20 (15.4)	29 (15.2)	43 (26.5)	2 (4.8)	-
	TAMU-Q (Engineering)	19 (4.8)	5 (1.9)	14 (10.8)	10 (5.2)	7 (4.3)	2 (4.8)	-
	CMU-Q (Biology, IT)	77 (19.4)	44 (16.9)	32 (24.6)	39 (20.4)	28 (17.3)	10 (23.8)	-
	GU-Q (Politics, History)	38 (9.6)	35 (13.4)	3 (2.3)	32 (16.8)	6 (3.7)	0 (0.0)	-
	NU-Q (Communication)	35 (8.8)	24 (9.2)	10 (7.7)	18 (9.4)	14 (8.6)	3 (7.1)	-
	UCL-Q (Information)	5 (1.3)	4 (1.5)	1 (0.8)	2 (1.0)	2 (1.2)	1 (2.4)	-
	ABP (pre-university)	14 (3.5)	8 (3.1)	6 (4.6)	0 (0.0)	3 (1.9)	11 (26.2)	-
	HBKU (Multidisciplinary)	48 (12.1)	23 (8.8)	24 (18.5)	9 (4.7)	32 (19.8)	7 (16.7)	-
	QF (Qatar Foundation)	18 (4.6)	12 (4.6)	6 (4.6)	2 (1.0)	14 (8.6)	2 (4.8)	-
	Other (various)	15 (3.8)	10 (3.8)	5 (3.8)	6 (3.1)	6 (3.7)	3 (7.1)	-
	Missing	5 (1.3)	2 (0.8)	2 (1.5)	0 (0.0)	4 (2.5)	0 (0.0)	-

5 participants did not report gender, 1 participant did not report role, 5 participants did not report institution

Responses to a first series of questions about the Zika virus are given in Table 2 and presented for all participants, and then separately for females and for males, for students, staff, faculty members and for responders to the online survey. Response frequency to the specific questions follows:

**Table 2:** Responses to the survey: General knowledge about Zika virus

Question	Answer	All	Female	Male	Student	Staff	Faculty	Online survey
		*N* (% col)	*N* (% col)	*N* (% col)	*N* (% col)	*N* (% col)	*N* (% col)	*N* (% col)
	All participants	446 (100)	280 (100)	161 (100)	191 (100)	162 (100)	42 (100)	50 (100)
How did you hear about Zika virus?	I have not heard about Zika virus	68 (15.2)	48 (17.1)	18 (11.2)	44 (23.0)	15 (9.3)	4 (9.5)	4 (8.0)
	Friends or family	75 (16.8)	46 (16.4)	27 (16.8)	38 (19.9)	25 (15.4)	5 (11.9)	7 (14.0)
	Doctor’s office	8 (1.8)	4 (1.4)	4 (2.5)	5 (2.6)	1 (0.6)	1 (2.4)	1 (2.0)
	Radio	48 (10.8)	27 (9.6)	21 (13.0)	14 (7.3)	18 (11.1)	8 (19.0)	8 (16.0)
	TV	191 (42.8)	110 (39.3)	81 (50.3)	66 (34.6)	83 (51.2)	23 (54.8)	19 (38.0)
	Internet	271 (60.8)	161 (57.5)	108 (67.1)	105 (55.0)	105 (64.8)	24 (57.1)	37 (74.0)
	Social media	195 (43.7)	115 (41.1)	78 (48.4)	83 (43.5)	76 (46.9)	15 (35.7)	21 (42.0)
	Another source	23 (5.2)	15 (5.4)	8 (5.0)	8 (4.2)	7 (4.3)	2 (4.8)	6 (12.0)
	I cannot remember the source	6 (1.3)	4 (1.4)	2 (1.2)	2 (1.0)	2 (1.2)	2 (4.8)	0 (0.0)
Is there a vaccine to prevent Zika?	Yes	97 (21.7)	68 (24.3)	26 (16.1)	49 (25.7)	30 (18.5)	7 (16.7)	11 (22.0)
	No [√]	306 (68.6)	182 (65.0)	122 (75.8)	123 (64.4)	117 (72.2)	31 (73.8)	34 (68.0)
	Missing	43 (9.6)	30 (10.7)	13 (8.1)	19 (9.9)	15 (9.3)	4 (9.5)	5 (10.0)
How is Zika spread?	Eating contaminated food	21 (4.7)	16 (5.7)	4 (2.5)	15 (7.9)	6 (3.7)	0 (0.0)	0 (0.0)
	Drinking polluted water	31 (7.0)	20 (7.1)	10 (6.2)	20 (10.5)	7 (4.3)	2 (4.8)	2 (4.0)
	Through sexual intercourse [√]	126 (28.3)	72 (25.7)	53 (32.9)	47 (24.6)	48 (29.6)	17 (40.5)	14 (28.0)
	Through coughing and sneezing	52 (11.7)	34 (12.1)	17 (10.6)	32 (16.8)	15 (9.3)	1 (2.4)	4 (8.0)
	Through breastfeeding	37 (8.3)	25 (8.9)	12 (7.5)	18 (9.4)	11 (6.8)	4 (9.5)	4 (8.0)
	From a blood transfusion [√]	103 (23.1)	64 (22.9)	39 (24.2)	40 (20.9)	43 (26.5)	12 (28.6)	8 (16.0)
	Mother to child during pregnancy [√]	192 (43.0)	127 (45.4)	64 (39.8)	75 (39.3)	84 (51.9)	19 (45.2)	14 (28.0)
	Bite from an infected mosquito [√]	327 (73.3)	201 (71.8)	123 (76.4)	124 (64.9)	124 (76.5)	34 (81.0)	45 (98.0)
	Other	4 (0.9)	2 (0.7)	1 (0.6)	1 (0.5)	3 (1.9)	0 (0.0)	0 (0.0)
Who can get Zika?	Adult men [√]	253 (56.7)	141 (50.4)	110 (68.3)	92 (48.2)	103 (63.6)	28 (66.7)	30 (60.0)
	Adult women [√]	269 (60.3)	152 (54.3)	115 (71.4)	101 (52.9)	110 (67.9)	28 (66.7)	30 (60.0)
	Pregnant women [√]	366 (82.1)	226 (80.7)	137 (85.1)	141 (73.8)	146 (90.1)	36 (85.7)	43 (86.0)
	Children [√]	304 (68.2)	182 (65.0)	118 (73.3)	122 (63.9)	117 (72.2)	31 (73.8)	33 (66.0)
If a pregnant woman has Zika, there are risks for her baby/fetus	Agree [√]	345 (77.4)	214 (76.4)	127 (78.9)	135 (70.7)	136 (84.0)	37 (88.1)	36 (72.0)
	Disagree	3 (0.7)	1 (0.4)	2 (1.2)	1 (0.5)	2 (1.2)	0 (0.0)	0 (0.0)
	Not sure	98 (22.0)	65 (23.2)	32 (19.9)	55 (28.8)	24 (14.8)	5 (11.9)	14 (28.0)
General knowledge about Zika^a^	POOR	295 (66.1)	199 (71.1)	91 (56.5)	143 (74.9)	99 (61.1)	23 (54.8)	29 (58.0)
	BASIC	122 (27.4)	62 (22.1)	60 (37.3)	41 (21.5)	48 (29.6)	15 (35.7)	18 (36.0)
	BROAD	29 (6.5)	19 (6.8)	10 (6.2)	7 (3.7)	15 (9.3)	4 (9.5)	3 (6.0)

[√] indicates correct answers; Multiple answers could be selected for the first, third and fourth questions

^a^ **POOR**: Don’t know that there is currently no vaccine for Zika virus and/or that the disease is transmitted by infected mosquitoes and/or that anyone could get Zika virus; **BASIC:** Know that there is currently no vaccine for Zika virus, that the disease is transmitted by infected mosquitoes and that anyone could get Zika virus; **BROAD:** In addition, know that the disease could be transmitted through sexual intercourse, blood transfusion or during pregnancy, and that if a pregnant woman has Zika virus, there are risks for her baby/fetus (All 10 correct answers checked)

Sixty eight (15.2%) participants never heard about Zika virus. The most common source of information for those who heard about Zika virus is internet (60.8%), followed by social media (43.7%), television (42.8%), friends or family (16.8%), radio (10.8%) or doctor's office (1.8%).

Ninety seven (21.7%) participants thought that a vaccine to prevent Zika virus exists, while 9.6% did not answered this question. Majority (68.6%) knew that no vaccine is currently available.

Bite from an infected mosquito (73.3%) was the most frequently reported source of infection with the Zika virus. 192 (43.0%) participants knew that Zika virus could be transmitted from mother-to-child during pregnancy, 126 (28.3%) through sexual intercourse and 103 (23.1%) from a blood transfusion. Against the current evidence, 52 (11.7%) participants thought that Zika virus could also be spread through coughing and sneezing, 37 (8.3%) through breastfeeding, 31 (7.0%) drinking polluted water and 21 (4.7%) eating contaminated food.

Three hundred and sixty six (82.1%) participants thought that Zika virus could be transmitted to pregnant women, 304 (68.2%) to children, 269 (60.3%) to adult women and 253 (56.7%) to adult men. In addition, 345 (77.4%) participants knew that if a pregnant woman has Zika virus, there are risks for her baby or fetus. Ninety eight (22.0%) participants were uncertain about this last question and only 3 (0.7%) disagreed.

### General knowledge about Zika virus

When we summarized responses to all items related to the general knowledge about Zika virus, only 29 (6.5%) participants demonstrated to have a “*broad*” general knowledge about Zika virus (i.e. responded correctly to 10 questions about Zika virus), 122 (27.4%) a “*basic*” knowledge and 295 (66.1%) no or only “*poor*” knowledge about Zika virus.

### Factors associated with general knowledge about Zika virus

At univariate analysis, males (OR = 1.89, 95% CI 1.26–2.83), older participants (OR = 2.61, 95% CI 1.37–4.99 for age 30–39 and OR = 3.45, 95% CI 1.75–6.79 for age 40+ compared to those aged less than 20 years), those originating from Asia or Africa (OR = 1.81, 95% CI 1.07–3.09) or Europe, North America, Australia or New Zealand (OR = 4.65, 95% CI 2.67–8.08) compared to those originating from GCC or other Middle East countries, staff (OR = 1.91, 95% CI 1.21–3.01), faculty members (OR = 2.48, 95% CI 1.24–4.94) or responders to the online survey (OR = 2.17, 95% CI 1.13–4.16) compared to students and those at a school of medicine (WCM-Q) (OR = 1.51, 95% CI 0.90–2.52) were more likely to have higher general knowledge about Zika virus, while those at an artistic school (VCU-Q) (OR = 0.13, 95% CI 0.04–0.41) had lower general knowledge about Zika virus (Table 3). At multivariable analysis country of origin (Europe, North America, Australia or New Zealand OR = 3.93, 95% CI 2.11–7.34), attending, working or teaching at a school of medicine (OR = 1.81, 95% CI 1.03–3.20) were associated with higher general knowledge about Zika virus, while attending, working or teaching at an artistic school (OR = 0.19, 95% CI 0.06–0.67) were associated with lower general knowledge about Zika virus (Table 3). The association with age, gender and study groups lost statistical significance after adjustment for nationality and institutions.

**Table 3:** Predictors of general knowledge about Zika virus at univariate and multivariable analysis

Variable	Category	POOR Knowledge	BASIC or BROAD knowledge about Zika				
				Univariate analysis		Multivariable analysis	
		*N* (% row)	*N* (% row)	OR (95% CI)	*P*-value	OR (95% CI)	*P*-value
Gender	Female	199 (71.1)	81 (28.9)	1.00		1.00	
	Male	91 (56.5)	70 (43.5)	**1.89 (1.26–2.83)**	**0.002**	1.42 (0.90–2.24)	0.13
Age	Less than 20	69 (78.4)	19 (21.6)	1.00		1.00	
	20–29	115 (70.6)	48 (29.4)	1.52 (0.82–2.79)	0.18	0.92 (0.45–1.85)	0.81
	30–39	57 (58.2)	41 (41.8)	**2.61 (1.37–4.99)**	0.004	1.06 (0.40–2.80)	0.91
	40 or more	39 (51.3)	37 (48.7)	**3.45 (1.75–6.79)**	0.0004	1.02 (0.37–2.85)	0.96
Marital status	Single	178 (69.5)	78 (30.5)	1.00			
	Ever married	113 (61.7)	70 (38.3)	1.41 (0.95–2.11)	0.09		
Nationality	GCC countries	53 (81.5)	12 (18.5)	1.00		1.00	
	Middle East	60 (78.9)	16 (21.1)				
	Asia	90 (68.7)	41 (31.3)	**1.81 (1.07–3.09)**	**0.03**	1.69 (0.96–2.95)	0.07
	Africa	21 (70.0)	9 (30.0)				
	Europe	19 (57.6)	14 (42.4)	**4.65 (2.67–8.08)**	**<0.0001**	**3.93 (2.11–7.34)**	**<0.0001**
	North America/Australia/New Zealand	34 (42.0)	47 (58.0)				
Role	Student	143 (74.9)	48 (25.1)	1.00		1.00	
	Staff	99 (61.1)	63 (38.9)	**1.91 (1.21–3.01)**	**0.005**	1.02 (0.50–2.08)	0.96
	Faculty	23 (54.8)	19 (45.2)	**2.48 (1.24–4.94)**	**0.01**	1.14 (0.43–3.06)	0.79
	Online survey	29 (58.0)	21 (42.0)	**2.17 (1.13–4.16)**	**0.02**	1.51 (0.65–3.52)	0.34
Institution	Other institutions	179 (66.5)	90 (33.5)	1.00		1.00	
	VCU-Q (Art, design)	45 (93.8)	3 (6.3)	**0.13 (0.04–0.41)**	**0.0006**	**0.19 (0.06–0.67)**	**0.04**
	WCM-Q (Medicine)	41 (55.4)	33 (44.6)	1.51 (0.90–2.52)	0.07	**1.81 (1.03–3.20)**	**0.009**

Odds ratio (OR) and 95% confidence intervals (CI) obtained from logistic regression model. All variables significantly associated with knowledge about Zika virus at univariate analysis were included in the multivariable model

Bold data refers to findings significant at *P*  <  0.05

### Personal perception about Zika virus

Responses to a subsequent series of questions about the personal perception of participants about Zika virus are given in Table 4 **,** presented for all participants, and stratified according to the general knowledge about Zika virus assessed from the previous set of questions.

**Table 4:** Responses to the survey: Personal perception about Zika virus and travel to endemic country

Question	Answer	All	General knowledge about Zika			*P*-value
		subjects	POOR	BASIC	BROAD	
		*N* (%)	*N* (%)	*N* (%)	*N* (%)	
Total	N (%)	446 (100)	295 (100)	122 (100)	29 (100)	
I am knowledgeable about the risk of Zika virus	Agree	102 (22.9)	45 (15.3)	41 (33.6)	16 (55.2)	
	Disagree	114 (25.6)	85 (28.8)	29 (23.8)	0 (0.0)	
	Not sure	230 (51.6)	165 (55.9)	52 (42.6)	13 (44.8)	<0.0001
I can control my exposure to Zika virus	Agree	140 (31.4)	77 (26.1)	50 (41.0)	13 (44.8)	
	Disagree	55 (12.3)	32 (10.8)	20 (16.4)	3 (10.3)	
	Not sure	251 (56.3)	186 (63.1)	52 (42.6)	13 (44.8)	0.002
The thought of Zika virus makes me feel anxious/worried	Agree	140 (31.4)	98 (33.2)	31 (25.4)	11 (37.9)	
	Disagree	102 (22.9)	56 (19.0)	41 (33.6)	5 (17.2)	
	Not sure	204 (45.7)	141 (47.8)	50 (41.0)	13 (44.8)	0.02
Zika can be treated	Agree	96 (21.5)	67 (22.7)	27 (22.1)	2 (6.9)	
	Disagree	91 (20.4)	39 (13.2)	39 (32.0)	13 (44.8)	
	Not sure	259 (58.1)	189 (64.1)	56 (45.9)	14 (48.3)	<0.0001
Zika can be prevented	Agree	225 (50.4)	137 (46.4)	74 (60.7)	14 (48.3)	
	Disagree	24 (5.4)	12 (4.1)	10 (8.2)	2 (6.9)	
	Not sure	197 (44.2)	146 (49.5)	38 (31.1)	13 (44.8)	0.01
Exposure to Zika virus always leads to sickness	Agree	108 (24.2)	78 (26.4)	21 (17.2)	9 (31.0)	
	Disagree	104 (23.3)	50 (16.9)	44 (36.1)	10 (34.5)	
	Not sure	234 (52.5)	167 (56.6)	57 (46.7)	10 (34.5)	0.0002
In the past year, have you traveled to any of the countries listed below?	Yes	22 (4.9)	13 (4.4)	7 (5.7)	2 (6.9)	0.75

Not sure: Neutral / Don’t know / Missing

One hundred and two (23.1%) participants recognized to be knowledgeable about the risk of Zika virus, 114 (25.6%) disagree and 230 (51.6) were not sure. Participants’ self perception correlated with their actual knowledge about Zika virus: 57 (55.9%) of the 102 participants who reported being knowledgeable about the risk of Zika virus have a “*basic*” or “*broad*” knowledge about Zika virus, against 29 (25.4%) of 114 who reported not being knowledgeable about the risk of Zika virus and 65 (28.3%) of 230 with neutral response (*P* < 0.0001).

In total, 45 (10.1%) participants reported to be knowledgeable but actually had a “*poor*” knowledge about Zika virus. The frequency of participants with such an erroneous perception of knowledge about the risk of Zika virus was not associated with gender, age, nationality, marital status, education or participants group. It was however significantly higher (25.0%) among the 48 participants from artistic school (VCU-Q) (*P* < 0.0001).

In response to the last questions, 140 (31.4%) participants thought that “*they can control their exposure to Zika virus*”, 140 (31.4%) reported that “*the thought of Zika virus makes them feel anxious or worried*”, 96 (21.5%) thought that “*Zika virus can be treated*”, 225 (50.4%) that “*Zika virus could be prevented*” and 108 (24.2%) that “*exposure to Zika virus always leads to sickness*” (Table 4).

Only 22 (4.9%) participants reported having travelled to any of the listed countries, where Zika virus is endemic, in the past year.

## Discussion

The aim of this study was to determine the level of knowledge about Zika virus--an emerging potentially serious viral infection in a sample of educated persons living in Qatar, one of the GCC nations. Understanding the level of disease knowledge in the general population is an important public health measure for prevention or control of any disease. Although not a region where the disease is prevalent, there are many flights from the Middle East to countries or areas where the disease is widespread, raising the real possibility that returning passengers who have contracted Zika virus, could unknowingly transmit this disease. Returning Zika virus-infected patients have already been documented in the Middle East [9].

Our survey was designed to capture basic knowledge covering areas such as mode of disease transmission, disease prevention, susceptible populations, and availability of a protective vaccine. We also included several self-assessment questions to determine if there was a correlation between respondents actual knowledge and their perception of what they knew about this disease.

Although not a representative population sample, we believe that the education level of the respondents is higher than the background population, implying that the knowledge level about Zika virus in the general population is likely to be lower than in our sample. We have no information about Zika virus awareness in regional health care workers, but in the sample of medical students the responses indicated that about a third of this group were poorly informed and none had a broad knowledge of basic facts about Zika virus.

We carried out the study at about the time of the 2016 Olympics in Brazil, a period when there was great international interest about the threat of Zika virus to athletes and to travelers. Despite the widespread global media interest at the time of the survey, the questionnaire revealed that many respondents are unaware of critical information concerning Zika virus. Fifteen percent of respondents had never heard of Zika virus, 27% were unaware that mosquitoes were involved in disease transmission, and 22% believed there is already a protective vaccine. Another disturbing finding is that of the 102 respondents who considered themselves knowledgeable about Zika virus, the survey revealed that nearly half proved to be poorly informed. These findings indicate a need for disseminating information about this disease, especially for persons who will be travelling to areas where the disease is prevalent.

In multivariable analysis, respondents born in (Europe, North America, Australia and New Zealand) had superior knowledge about Zika virus than respondents born in other countries (*P* = 0.0001). We do not have a precise explanation for this difference, but expatriate students may be more knowledgeable about Zika than local students, since they would have been likely to be exposed to information about Zika from local sources when travelling to or from their native country. This should be explored further since it is important for public health reasons to understand why a local endemic population does not understand or know much about an emerging infection like Zika virus. Since there was no local outbreak of Zika in the country, the locals may not have been interested as such in learning about the Zika virus. Public health authorities can play a role in increasing awareness and disseminating information about emerging infections within the local population. In addition to student source, type of training was also a significant predictor: students, faculty or staff associated with a medical school performed significantly better (*P* = 0.009) than other groups, while the equivalent group at an artistic school did significantly worse (*P* = 0.04).

Is Zika virus a current threat to persons living in the Middle East? Although the prevalence is always likely to remain low, person to person transfer of infection could occur in one of two ways. The first method would be via mosquito transmission from an infected to an unaffected person. This is likely to be rare in this generally arid region, although two primary vector species (*Aedes albopictus* and *Aedes aegypti* mosquitoes) which can transmit Zika virus are inhabitants of the region [7, 8]. Another more likely pathway is person to person transfer of disease either by sexual contact or by other inadvertent exchange of body fluids [10, 11]. This could be a threat not only to family members but to health care workers caring for patients returning from a region with a high prevalence of Zika virus who have had or currently are ill with Zika virus. Blood or body fluids from Zika virus-infected patients may harbor the virus for up to 3 weeks and possibly longer [12]. Therefore, health care workers will need to be particularly vigilant when caring for persons who have recently returned from high risk regions.

An informed population is a key element in the control of infectious disease [13]. What is the best method to provide information about Zika virus control and prevention to the community? Our survey revealed that most respondents found out about Zika virus via the internet or social media. This form of communication is widely available in the Middle East and could be used to rapidly disseminate useful public health information about Zika virus.

Similar studies have been conducted during the same period including a cross sectional study conducted among 177 persons including interns, graduate staff and post graduate faculty in Luxmi Bai Institute of Dental Sciences, Patiala, India. In contrast to our results, all the participants in this study demonstrated adequate knowledge on the topic of Zika and the scores almost increased with proceeding age and education. This survey was however limited to health care professionals [14]. In another self-administered internet based survey, 442 members of doctor organizations in Aceh province, Indonesia, demonstrated relatively low knowledge about Zika infection. In this study only 35.9% of them had a good knowledge on Zika infection, which is comparable to that observed in our study from Qatar [15]. In a cross-sectional survey conducted in India among 412 private dental practitioners, high knowledge about Zika virus infection was only reported by 38.2% participants. Most of the knowledge was reported to come from television (37.8%) which is similar to our study from Qatar (42.8%). Dental practitioners reported knowledge from journals to be 4.7%, whereas our study from Qatar reported no knowledge from the category of journals [16].

The main study strength is that it reports on knowledge and self-assessment of knowledge about a significant emerging infectious disease from an educated sample of respondents in Qatar. Although not a representative sample of the Middle East, the response rate for the in-person survey was high (88.8%) and it is likely that a more representative sample of less well-educated persons from this region would have a lower knowledge level than observed in this sample. Another strength is that the survey included respondents from many different countries, thus providing regional comparative data about health knowledge for this disease. The main study limitation is the non-representative population sampling strategy. Since the in-person survey was administered only in Education City, Qatar, this data has local significance. However, we believe that information gathered from this privileged sample could be important to healthcare givers, identifying an area where health promotion and disease prevention programs could be established. Furthermore, even though this study is essentially single-site as may be the case for previously published studies, these findings can be extrapolated to other populations elsewhere.

## Conclusion

This study reveals a lack of the local population engaging with information about Zika virus--a potentially serious emerging infectious disease—in an educated sample of individuals residing in a Middle East Country to date. There have been few reported patients with Zika virus in this region, but there is the real potential for persons to contract the disease from previously infected persons returning to the region. Information programs could be especially important, especially for individuals planning to travel to any one of the many countries where Zika virus is now prevalent.

## Abbreviations

CI: Confidence intervals
GCC: Gulf Cooperation Council
OR: Odds ratio
